# Editorial: Women in science: translational research in rehabilitation

**DOI:** 10.3389/fresc.2023.1306030

**Published:** 2023-10-26

**Authors:** Ada Tang, Ada W. S. Leung

**Affiliations:** ^1^ School of Rehabilitation Science, McMaster University, Hamilton, ON, Canada; ^2^ Department of Occupational Therapy and Neuroscience and Mental Health Institute, University of Alberta, Edmonton, AB, Canada

**Keywords:** rehabilitation, women in science, women in STEM, sex and gender, editorial

**Editorial on the Research Topic** Women in science: translational research in rehabilitation

We are excited to have this platform in Frontiers to feature the *Women in Science* series in Frontiers in Rehabilitation Science: Translational Rehabilitation. In this series, we share research about women featuring studies led by women.

It is well established that women have been historically underrepresented in many areas of research (1–5) and do not reflect the disease prevalence of many health conditions. Factors associated with lower participation of women in research trials include trial-specific factors (women are often excluded from trials with limits on maximum age eligibility or presence of co-morbidities) and higher trial burden (women are more likely to report transportation barriers) (2). Under-representation can have significant consequences on outcomes, particularly when the body of evidence that is used to inform clinical practice is largely based on men and ignores inherent differences in health, illness, disease, and outcomes between men and women. Indeed, the resulting knowledge gap that remains has been powerfully described for women and cardiovascular disease as “five glaring ‘unders’”: under-aware, under-diagnosed, under-treated, under-researched, and under-supported (6). We believe these “unders” extend beyond cardiovascular disease, but to other populations as well.

There are known biological, physiological, and hormonal differences between sexes, and sociocultural factors associated with gender also influence health outcomes. Relevant to rehabilitation, women are less likely to engage in rehabilitation programs (7), often placing a lower priority on their own health to fulfill roles historically held by women such as caregiving and household responsibilities (8). Positively, however, when women do participate in rehabilitation, they benefit similarly to men (9–11).

In this special topic, *Women in Science: Translational Research in Rehabilitation*, we are proud to feature women in science as lead, contributing, and senior authors in this series. The articles featured in this series highlight women-led studies in a range of areas within rehabilitation science. Moreover, these women represent the full continuum along the research pipeline, from established scientists who are leaders in the field to trainees who are the future of rehabilitation science.

Two studies within this special series described challenges, opportunities, and rehabilitation outcomes observed in women. For example, Aranceta-Garza and Ross studied the functionality and efficacy of wrist-hand orthoses for healthy women, recognizing the need for quality assessment of assistive devices for women. Wiley et al. described sex-based differences in the relationship between walking and cognitive function after stroke. This special topic also featured research focused on the implementation of community-based exercise programs and clinical guidelines for stroke rehabilitation. Two studies conducted by Aravind, Bashir et al., Aravind, Graham et al. highlighted conditions that influence the sustainability of community-based exercise programs delivered through healthcare community partnerships for people with balance and mobility limitations as well as for people recovering from stroke. Dos Santos et al. conducted a review to identify and describe standardized tools for assessing balance and mobility in stroke, which helps develop clinical guidelines for stroke rehabilitation.

These studies take steps toward addressing five “unders” in rehabilitation research in women. They help contribute to an evidence base that helps counter the under-representation of women as participants in research. They help to inform future research to establish strategies to counter the under-treatment and under-support provided to women across a range of health conditions. Moreover, we feature studies that are led by women scientists who also represent diversity in experiences, cultures, languages, ethnicities, and educational backgrounds, which can help to broaden perspectives and approaches used in the conduct of research and interpretation of findings.

We applaud the women researchers who have led in this field, engaging in activities such as community outreach, public awareness, advocacy for change, and training and mentorship to develop the next generation of future leaders. We hope you enjoy reading these articles as much as we did putting this series together for you. Thank you for your interest in this special topic in Frontiers in Rehabilitation Science: Translational Rehabilitation for the *Women in Science* series. Our goal is that the articles featured in this series ignite further conversations, initiatives, and future research to promote and amplify the voices of women in science and its intersectionality with other historically represented groups, especially in rehabilitation research.
